# Optimum Antithrombotic Therapy in Patients Requiring Long-Term Anticoagulation and Undergoing Percutaneous Coronary Intervention

**DOI:** 10.1155/2018/5690640

**Published:** 2018-03-25

**Authors:** Nayan Agarwal, Dhruv Mahtta, Cecil A. Rambarat, Islam Elgendy, Ahmed N. Mahmoud

**Affiliations:** Department of Medicine, University of Florida, Gainesville, FL, USA

## Abstract

Management of patients on long-term anticoagulation requiring percutaneous coronary intervention is challenging. Triple therapy with oral anticoagulant and dual antiplatelet therapy is the standard of care. However, there is no strong evidence to support this strategy. There is emerging data regarding the safety and efficacy of dual therapy with oral anticoagulant and single antiplatelet therapy in these patients. In this comprehensive review we highlight available evidence regarding various antithrombotic regimens' efficacy and safety in patient with coronary artery disease undergoing percutaneous coronary intervention with long-term anticoagulation therapy requirements.

## 1. Background

Patients with mechanical heart valves, a prior systemic thromboembolic event and atrial fibrillation/flutter (AF), often require long-term anticoagulation [1, 2]. About 20% to 30% of these patients have concomitant ischemic heart disease requiring percutaneous coronary intervention and stent implantation (PCI) [3–5]. This would mandate the use of dual antiplatelet therapy (DAPT) (aspirin and an adenosine diphosphate antagonist) for prevention of stent thrombosis and adverse events following PCI [6]. It is often a clinical dilemma, whether to use dual therapy (DT) with either oral anticoagulant (OAC) and single antiplatelet therapy (SAPT) or DAPT or triple therapy (TT) with OAC and DAPT in these patients [7].

Although primary intent of TT is to decrease the incidence of major adverse cardiac events (MACE), especially stent thrombosis, it has been found to be associated with a high annual risk of bleeding [8, 9], which in turn is strongly associated with recurrent hospitalization and increased morbidity and mortality [10, 11]. Recently, new evidence has emerged questioning the benefit of TT and suggesting a regimen of dual therapy (DT) with OAC and a single antiplatelet (SAPT) agent might be equally efficacious to TT with a lower incidence of major bleeding [8, 9, 12]. There is also emerging evidence that use of DAPT in these patients is associated with similar outcomes to TT with less bleeding [13, 14]. Hence, we attempted to review the available evidence with regard to different antithrombotic regimes in patients on long-term OAC requiring PCI.

## 2. Pathophysiology of Thrombogenesis in AF and in Acute Coronary Syndrome/PCI Patients

AF is the most common indication for OAC in patients on OAC requiring PCI. AF significantly increases the risk of thromboembolism [1, 15]. The type of thrombus in AF is mainly fibrin rich where platelets play a smaller role [16, 17]. Loss of atrial contraction causes stasis of blood flow in left atrium. There is also increased local expression in the dysfunctional atrial endocardium of prothrombotic molecules, such as tissue factor [18] and Von Willebrand factor (VWF) [19]. This indicates that inhibition of coagulation remains the mainstay in preventing AF related thromboembolism.

The pathogenesis of coronary thrombosis in patients with coronary artery disease (CAD) and those undergoing PCI is considered to be largely platelet driven. Under normal circumstances the endothelium is antithrombotic by expressing inhibitors of platelet activation, like nitric oxide (NO) and prostacyclin (PGI2), coagulation inhibitors, like tissue factor pathway inhibitor, and heparin sulphate, in addition to tissue-type plasminogen activator promoting fibrinolysis. However, when superficial erosions occur, the endothelium is activated towards hemostasis, becoming prothrombotic with expression of VWF and plasminogen activator inhibitor-1, in addition to reduced expression of NO and PGI2 [20]. This promotes platelet activation, which in turn can activate coagulation on the platelet surface. This suggests that platelet inhibition is the mainstay for avoiding plaque rupture or coronary stent induced coronary thrombosis.

## 3. Antithrombotic Regimens

### 3.1. Triple Therapy

Aspirin has always been cornerstone in treating ACS and/or PCI, OAC is needed for stroke prevention in AF, mechanical heart valves, and previous thromboembolism, and a P2Y12 inhibitor is essential for prevention of stent thrombosis. Current American College of Cardiology (ACC)/American Heart Association (AHA) [6] and European Society of Cardiology [4] guidelines both recommend TT in patients with an indication for anticoagulation undergoing PCI. However, this approach may result in excess major bleeding, with rates of 2.2% within the first month and 4 to 12% within the first year of treatment [21]. These guidelines are based mostly upon observational trials and expert opinion due to the scarcity of randomized data. However, the guidelines emphasize that the treatment period should be as short as possible because of the increased bleeding risk over time. The ISAR-TRIPLE (Intracoronary Stenting and Antithrombotic Regimen-Testing of a 6-Week Versus a 6-Month Clopidogrel Treatment Regimen in Patients With Concomitant Aspirin and Oral Anticoagulant Therapy Following Drug-Eluting Stenting) [22] failed to show a benefit of 6 months of TT over 6 weeks with regard to composite of death, myocardial infarction, definite stent thrombosis, stroke, or major bleeding. An explanation for this finding may be that approximately one-half of all bleeding events occurred in the first 6 weeks after PCI, when both groups received the same therapy consisting of aspirin, clopidogrel, and OAC.

Most of the studies evaluating the role of TT in patients with an indication for OAC requiring PCI used warfarin as the OAC [9, 23–26]. Although not supported by robust clinical data, guidelines recommend a target international normalized ratio (INR) to be between 2 and 2.5 when warfarin is used [1, 6]. Data regarding newer oral anticoagulant agents (NOACs) in patients requiring OAC and undergoing PCI is scant. The dose of NOACs in TT is also debatable. The ATLAS-ACS-TIMI 46 (Anti-Xa Therapy to Lower Cardiovascular Events in Addition to Standard Therapy in Subjects with Acute Coronary Syndrome-Thrombolysis in Myocardial Infarction 51) [27] demonstrated that standard 20 mg dose of rivaroxaban when added to DAPT in ACS patients significantly increased bleeding. In ATLAS-ACS TIMI 51 trial [28] a very low dose of rivaroxaban (2.5 mg twice daily) was associated with lower rates of death from cardiovascular causes, myocardial infarction, and stroke compared to DAPT alone in ACS patients without an increase in fatal bleeding, but this low dose of 2.5 mg twice daily has not been studied for prevention of stroke in AF patients. The usage of apixaban for ACS was first explored in the Apixaban for Prevention of Acute Ischemic and Safety Events (APPRAISE) trial, which did not show a benefit with apixaban in addition to standard post-ACS treatment but resulted in significantly increased bleeding [29]. Dabigatran also increased the risk of bleeding when added to DAPT in ACS patient [30]. The ACC/AHA guidelines have not made any specific recommendations regarding NOACS. European guidelines [4] give NOACS the same level of recommendation as warfarin and suggest using lower dose (dabigatran 110 mg twice daily, rivaroxaban 15 mg daily, or apixaban 2.5 mg twice daily) for patients requiring TT.

Role of rivaroxaban was investigated in PIONEER-AF PCI (Open-Label, Randomized, Controlled, Multicenter Study Exploring Two Treatment Strategies of Rivaroxaban and a Dose-Adjusted Oral Vitamin K Antagonist Treatment Strategy in Subjects with Atrial Fibrillation who Undergo Percutaneous Coronary Intervention) [12] as triple therapy, which showed a rivaroxaban based strategy resulting in similar efficacy with less bleeding compared to warfarin based strategy. Most recently, efficacy of DT with dabigatran versus TT was assessed by a team of researchers from the RE-DUAL PCI (Randomized Evaluation of Dual Therapy with Dabigatran versus Triple Therapy Strategy with Warfarin in Patients with nonvalvular atrial fibrillation that have undergone PCI with Stenting) trial [31]. This was multicentered, randomized-control trial consisting of 2725 patients where the primary end point was major or clinically relevant nonmajor bleeding event while the secondary endpoint consisted of thromboembolic events. At their mean follow-up period of 14 months, the authors concluded the risk of bleeding being lower among the patients in the DT with dabigatran cohort as compared to patients who received TT. DT with dabigatran was also shown to be noninferior to TT with regard to thromboembolic events. Future randomized studies such as Rivaroxaban and Ticagrelor in Atrial Fibrillation (RT-AF) [32] and Apixaban in Non-Valvular Atrial Fibrillation with a Recent Acute Coronary Syndrome or Undergoing Percutaneous Coronary Intervention (AUGUSTUS Trial) (NCT02415400) will provide more information on this subject.

There is paucity of data with regard to newer antiplatelet agents (ticagrelor and prasugrel) in combination with OAC. There are concerns that it can cause more bleeding compared to clopidogrel [50]. However, such speculations regarding the superiority or even inferiority of clopidogrel versus newer antiplatelet agents have yet to be proven by clinical data from randomized controlled trials. Nonetheless, at present both ACC/AHA and ESC guidelines recommend using clopidogrel, when TT is required.

### 3.2. Dual Therapy with OAC and SAPT

There are several studies that evaluated the role of DT of OAC and SAPT with TT [9, 12, 24–26, 33–42]. The WOEST [9] (what is the optimal antiplatelet and anticoagulant therapy in patients with oral anticoagulation and coronary stenting) was the first randomized study which tested the hypothesis of using DT with OAC and SAPT in patients with an indication for long-term OAC undergoing PCI. The combination of conventional therapy of warfarin, aspirin, and clopidogrel was tested against the combination of warfarin and clopidogrel over 1 year in 573 patients. DT arm had significantly less bleeding compared to TT (19.4% versus 44.4%, HR 0.36, *p* < 0.0001) and need for transfusion (3.9% versus 9.5%, OR 0.39, *p* = 0.011). The secondary end points of death, MI, stroke, and stent thrombosis were lower in DT compared to TT but did not reach statistical significance. In PIONEER-AF [12] the DT of low dose rivaroxaban (15 mg daily) plus P2Y12 inhibitor resulted in less overall bleeding (16.8% versus 26.7%, HR 0.59, *p* < 0.001) and bleeding requiring medical attention (14.6% versus 22.6%, HR 0.61, *p* < 0.001) without any significant difference in the composite of cardiovascular death, MI, or stroke. The P2Y12 inhibitor was clopidogrel in 85% of cases. Danish group [36] published results of a real-life nationwide retrospective registry of 12,165 patients which showed that after 1 year, there was no increased risk of recurrent coronary events for DT (HR: 0.69; 95% CI: 0.48 to 1.00) relative to TT, and bleeding risk was also nonsignificantly lower for warfarin plus clopidogrel (HR: 0.78, 95% CI: 0.55 to 1.12). Moreover, there was a similar risk for all-cause mortality in patients treated with warfarin plus clopidogrel as well as those receiving triple therapy, but the combinations of warfarin plus aspirin and aspirin plus clopidogrel were associated with a significant increase in all-cause mortality compared with triple therapy. Recent meta-analyses [8, 48] of these observational and randomized studies have also demonstrated a similar result with less bleeding with DT without a statistically significant difference in ischemic or thromboembolic end points.  Table 1 illustrates the baseline characteristics of trials comparing DAPT with TT following PCI.

As noticed in Table 1, the OAC was warfarin in majority of the studies, except for PIONEER, where rivaroxaban was used. Antiplatelet in the DT arm was clopidogrel in majority of studies, though aspirin was also used in several observational studies. Karjalainen et al. [42] demonstrated that combination of warfarin and aspirin resulted in more strokes and stent thrombosis compared to combination of warfarin and clopidogrel. Similarly in a network meta-analysis Liu et al. [46] demonstrated that the combination of OAC and clopidogrel was superior to OAC and aspirin with regard to major adverse cardiac events, stroke, MI, and all-cause mortality. Similar results were also seen in Danish registry [36] as discussed above. Newer antiplatelets (ticagrelor and prasugrel) were used only in about 15% of patients in PIONEER-AF; hence there is insufficient data regarding their use at this time.  Table 2 illustrates outcomes of DAPT compared with TT following PCI.

The ESC guidelines [1] recommend an initial 4-week-6-month TT based on CHADVASC and HAS-BLED score and then DT of OAC (warfarin or NOAC) and clopidogrel. WOEST [9] trial noticed that most bleeding episodes happened during the initial 180 days after PCI. Similar time frames for increased bleeding were seen in other studies of antiplatelet therapies [51, 52]. Hence, omission of the initial 4-week-6-month TT can result in reduction in bleeding as evidenced by WOEST [9] and PIONEER-AF [12]. In Table 3, we summarize the current guidelines pertaining to the use of DT and TT as recommended by ACC/AHA and ESC [4, 6, 43–45].

### 3.3. Dual Therapy with DAPT

DAPT is the cornerstone of therapy in patients with ACS and/or PCI. Aspirin alone has shown to reduce the incidence of stroke by 22% in AF [53, 54]. Can DAPT provide enough benefit in stroke protection? This hypothesis was tested in ACTIVE-W study [55] (Atrial fibrillation Clopidogrel Trial with Irbesartan for prevention of Vascular Events), which showed that, compared to warfarin only, the combination of aspirin and clopidogrel resulted in a significantly increased relative risk of 1.44 (1.18–1.76; *p* = 0.0003) of composite of stroke, systemic embolus, MI, or vascular death in patients with AF. Hence, DAPT is not considered effective for thromboembolic-prophylaxis in AF.

There are conflicting results on efficacy of DAPT in this cohort. A recent analysis published from National Cardiovascular Data Registry (NCDR) examined outcomes with DAPT versus TT in 4,959 patients > 65 years of age with acute MI and AF who underwent PCI [14]. Relative to DAPT, patients on TT had a similar risk of major adverse cardiac events (adjusted hazard ratio [HR]: 0.99 [95% CI: 0.86 to 1.16]), nonsignificantly lower risk of ischemic stroke (HR 0.66, 95% CI: 0.41–1.06), but significantly greater risk of bleeding requiring hospitalization (adjusted HR: 1.61 [95% CI: 1.31 to 1.97]), and greater risk of intracranial hemorrhage (adjusted HR: 2.04 [95% CI: 1.25 to 3.34]). In another single center retrospective study by Choi et al. [13], it was seen that, compared to TT, DAPT had less bleeding and no difference in composite of stroke, MI, or cardiac death. Danish registry study [36], Karjalainen et al. [42], Gao et al. [39], and Rubboli et al. [37] showed a higher risk of ischemic stroke, all-cause death, and major adverse cardiac events with DAPT compared to TT and OAC plus SAPT. The NCDR analysis [14] and results of Choi et al. [13] were underpowered to detect a difference in stroke and patients had less risk factors for stroke, which could undermine the benefits of TT compared to DAPT. Network meta-analysis by Liu et al. [46] demonstrated that DAPT was associated with worse outcomes compared with TT and OAC plus clopidogrel in this patient population.  Table 4 summarizes the results of various meta-analyses comparing DAPT with TT.

## 4. Further Considerations for Treatment Strategies

Based on the review of available evidence, combination of OAC and clopidogrel and TT are most efficacious. Combination of OAC and clopidogrel results in less bleeding compared to TT. Several large meta-analyses (Table 3) have suggested that OAC + SAPT either resulted in lower incidence or had equivalent rates of MACE when compared to TT. DAPT and combination of OAC and aspirin are inadequate with higher incidence of stroke, MI, and stent thrombosis.

Major bleeding events and blood transfusions have been associated with increased risk of death in patients undergoing PCI [10, 56]. Majority of the patients with indication for OAC needing PCI are older [8, 56], who have higher thromboembolic risk, higher bleeding risk, and significant comorbidities [57]. Effect of nonmajor bleeding should not be underestimated in these patients, since even superficial or “nuisance” bleeding can lead to discontinuation of antiplatelet therapy, which may lead to subsequent thrombotic complications such as stent thrombosis [56, 58]. Hence, a strategy to reduce bleeding is even more important in these patients.

There are several questions that still remain. Firstly, what is the optimal therapy for patients undergoing PCI who are treated with OAC and clopidogrel, once clopidogrel is discontinued? Do these patients require OAC plus aspirin or OAC alone after discontinuation of P2Y12 inhibitor? Optimal treatment strategy in this scenario is currently unknown and the lack of randomized trials assessing this troubles the daily clinician. Two large registry based studies—Danish registry [59] and ORBIT-AF (Outcomes Registry for Better Informed Treatment of Atrial Fibrillation) [60]—demonstrated that, compared to warfarin monotherapy, combination of warfarin and aspirin was associated with higher bleeding risk without reducing ischemic end points. European guidelines recommend long-term therapy with OAC only (Class 1, LOE B) with combination of OAC and SAPT in selected patients, for example, left main PCI, bifurcation PCI, and recurrent MI (Class 2b, LOE C). Secondly, data regarding role of NOACs in TT or DT is insufficient. PIONEER-AF showed rivaroxaban was superior to warfarin. Future trials like RT-AF and AUGUSTUS will provide more data on this subject. Thirdly, the role of newer P2Y12 inhibitors like ticagrelor and prasugrel is also unclear due to lack of studies using the combination of OAC plus ticagrelor/prasugrel.

## 5. Conclusions

The efficacy of TT in patients on OAC needing PCI has never been proven. This combination increases bleeding risk, which can result in adverse patients' outcomes. New evidence, from randomized controlled trial, nationwide registries, observational studies, and meta-analysis, indicates the great potential of the combination of OAC and clopidogrel without aspirin to improve clinical outcomes in comparison with triple therapy. Therefore, OAC combined with clopidogrel seems to be a reasonable alternative to triple therapy in patients on long-term OAC who undergo PCI.

## Figures and Tables

**Table 1:** Baseline characteristics of trials comparing dual therapy with triple therapy after PCI.

Study/author	Design	Year	Number of patients	Male (%)	TT	DT	Follow-up (months)	Indication for PCI	INR	Indication for anticoagulation
RE-DUAL PCI [31]	RCT	2017	2725	76%	w + a + c, w + a + t	d^*∗*^ + (c or t) [44%], d + (c or t) [56%]	14	ACS, CAD	2.0–3.0	AF

De Vecchis et al. [33]	R^*∗*^	2016	98	45%	w + a + c	w + c [NR], w + a [NR]	12	ACS, CAD	NR	AF, mechanical valve, VTE, dilated cardiomyopathy

PIONEER [12]	RCT	2016	1415	74%	w + a + c [96%], w + a + p [1%], w + a + t [3%]	R + c [93%], R + p [2%], R + t [5%]	12	ACS, CAD	2.0–3.0	AF

ORBIT-AF [34]	P^*∗*^	2016	1827	72%	w + a + c, w + a + p, d + a + c, d + a + p	w + a [NR], w + c [NR], w + p [NR], d + a [NR], d + c [NR], d + p [NR]	24	CAD	NR	AF

AFCAS [24]	P^*∗*^	2014	914	70%	w + a + c	w + c [100%]	12	ACS, CAD	1.8–3	AF

WARSTENT [25]	P^*∗*^	2014	401	26%	w + a + c	w + c [NR], w + a [NR]	12	ACS, CAD	1.8–4.5	AF, apical thrombus, apical akinesis, VTE, mechanical valve

Braun et al. [35]	R^*∗*^	2015	266	77%	w + a + c	w + t [100%]	3	ACS	2.0–3.0	AF, apical thrombus, apical akinesis, VTE, mechanical valve

Lamberts et al. [36]	P^*∗*^	2013	12165	61%	w + a + c	w + c [27%], w + a [73%]	12	ACS, CAD	NR	AF

WOEST [9]	RCT	2013	573	80%	w + a + c	w + c [100%]	12	ACS, CAD	2	AF, mechanical valve, VTE, apical aneurysm, PAD, EF < 30%

Rubboli et al. [37]	P^*∗*^	2012	632	73%	w + a + c	w + a [100%]	12	ACS, CAD	NR	AF, VTE, mechanical valve, dilated cardiomyopathy, ischemic heart disease, cardiac thrombus, CVA, LV aneurysm, biological heart valve

Persson et al. [38]	R^*∗*^	2011	1177	76%	w + a + c	w + c [45%], w + a [55%]	12	ACS	NR	NR

Gao et al. [39]	P^*∗*^	2010	622	71%	w + a + c	w + c [87%] or w + a [13%]	12	ACS, CAD	1.8–2.5	AF

MUSICA [40]	P^*∗*^	2009	405	81%	w + a + c, LMWH + a + c	w + c [80%], LMWH + c [4%], w + a [13%], LMWH + a [2%]	6	ACS, CAD	NR	AF, mechanical valve, CVA

Sørensen et al. [41]	R^*∗*^	2009	40812	63%	w + a + c	w + c [0.5%], w + a [2%]	18	ACS	NR	NR

GRACE [26]	P^*∗*^	2007	800	70%	w + a + c	w + c [51%], w + a [49%]	6	ACS	NR	AF, STEMI, VTE, mechanical valve

Karjalainen et al. [42]	R^*∗*^	2007	239	74%	w + a + c	w + c [58%], w + a [42%]	12	ACS, CAD	2–2.5	AF, mechanical valve, VTE, CVA

a = aspirin; ACS = acute coronary syndrome; AF = atrial fibrillation; c = clopidogrel; CAD = coronary artery disease; CVA = cerebral vascular accident; d = dabigatran (110 mg BID); d^*∗*^ = dabigatran (150 mg BID); DT = dual therapy; LMWH = low molecular weight heparin; NR = not reported; p = prasugrel; P^*∗*^ = prospective trial; PAD = peripheral artery disease; R = rivaroxaban; R^*∗*^ = retrospective trial; RCT = randomized-control trial; t = ticagrelor; TT = triple therapy; VTE = venous thromboembolism.

**Table 2:** Outcomes with dual therapy compared with triple therapy after PCI.

Study/author	MACE (%) [*p* value]	Mortality (%) [*p* value]	Stent thrombosis (%) [*p* value]	Total bleeding (%) [*p* value]	Major bleeding (%) [*p* value]
RE-DUAL PCI [31]	NR	4.9/5.6 [0.56]^*∗*^ 4.6/3.9 [0.44]^*∗∗*^	0.8/1.5 [0.15] 0.9/0.9 [0.98]	42.9/27.1 [<0.001] 41.4/33.3 [<0.001]	9.2/5.0 [<0.001] 8.4/5.6 [0.02]
De Vecchis et al. [33]	27.1/12.9 [0.32]	8.3/0 [0.26]	2/0 [0.59]	16.7/19.4 [0.90]	8.3/6.5 [0.89]
PIONEER [12]	6.0/6.5 [0.75]	1.9/2.4 [0.52]	0.7/0.8 [0.79]	26.7/16.8 [<0.01]	3.3/2.1 [0.23]
ORBIT-AF [34]	NR	4.1/5.4 [0.57]	NR	NR	5.68/5.85 [0.66]
AFCAS [24]	22/18 [0.72]	11/7 [0.54]	1/3 [0.60]	18/16 [0.66]	10/7 [0.43]
WARSTENT [25]	16/15 [0.98]	5/0 [0.45]	1/0 [0.76]	11/5 [0.34]	4/5 [0.84]
Braun et al. [35]	NR	3.2/3.8 [NS]	0/0 [NS]	NR	7/7.5 [NS]
Lamberts et al. [36]	NR	8.9/7.1 [NS]	NR	14.3/10.9 [NS]	0.9/0.5 [NS]
WOEST [9]	NR	6.3/3.5 [0.03]	3.2/1.4 [0.17]	44.4/19.4 [<0.01]	5.6/3.2 [0.16]
Rubboli et al. [37]	32/24.6 [0.19]	9.9/10.2 [0.78]	2.7/2.0 [0.77]	NR	5.0/2.6 [0.32]
Persson et al. [38]	NR	3.0/4.2 [0.43]	NR	4.7/1.3 [0.02]	2.7/0.3 [0.03]
Gao et al. [39]	8.8/14.9 [0.01]	4.4/5.8 [0.17]	0.7/1.7 [0.73]	11.8/7.4 [0.038]	2.9/2.5 [0.73]
MUSICA [40]	23.7/26.1 [0.001]	6.8/10.9 [0.06]	4.0/8.7 [0.04]	15.5/13 [0.02]	4.3/6.5 [0.29]
Sørensen et al. [41]	NR	[NS]	NR	3.2/1.6 [NS]	NR
GRACE [26]	NR	5.1/6.5 [0.47]	NR	NR	5.9/4.6 [0.46]
Karjalainen et al. [42]	21.9/11 [0.003]	8.7/1.8 [0.003]	4.1/1.3 [0.09]	NR	8.2/2.6 [0.01]

NR = not reported; NS = statistically nonsignificant; number preceding “/” denotes TT (triple therapy) and number proceeding “/” denotes DT (dual therapy), TT/DT; for RE-DUAL PCI: TT/DT^*∗*^ = Therapy with Dabigatran 110 mg BID; TT/DT^*∗∗*^ = Therapy with Dabigatran 150 mg BID.

**Table 3:** Guideline recommendations regarding triple therapy.

Class of evidence	2016 European Society of Cardiology Guidelines on AF [43]	2014 European Consensus on AF and PCI [4]	2016 ACC/AHA Guideline Focused Update on Duration of Dual Antiplatelet Therapy in Patients With Coronary Artery Disease [6]	2014 ACC/AHA Guidelines on NSTEMI [44]	2013 ACC/AHA Guidelines on STEMI [45]
Summary and synthesis of guideline, expert consensus documents, and comprehensive review article recommendations			(1) Keep TT duration as short as possible (2) Consider target INR 2.0–2.5 when warfarin is used as part of TT (3) Clopidogrel is the P2Y12 inhibitor of choice (4) PPIs should be used in patients with history of GI bleeding and those who are at high risk of bleeding while being on TT		

(I)	(1) After ACS or PCI: OAC monotherapy after initial 12 months (2) After elective PCI: OAC monotherapy after initial 6 months in patients with high bleeding risk			(1) Minimize duration of TT to limit risk of bleeding (2) Addition of PPI therapy in patients with prior history of GI bleeding who are started on TT	

(IIa)	(1) Stable CAD with elective PCI: 1 month of TT (2) Stenting after ACS: 1–6 months of TT (3) ACS without stenting: up to 12 months of DT (4) In general, minimize duration of TT and after completion of TT, DT until 12 months after PCI or ACS	(1) Stable CAD with elective PCI: 1–6 months of TT (2) ACS: 1–6 months of TT (3) After completion of TT, DT until 12 months after PCI or ACS (4) Consider lower iNR goal for warfarin (2.0–2.5) when part of TT		Consider addition of PPI therapy in patients without prior history of GI disturbances who are started on TT	

(IIb)	DT with OAC + clopidogrel may be considered as an alternative therapy in selective patients	(1) DT with OAC + clopidogrel may be considered as an alternate to TT in selected patients (2) ACS: 6–12 months of TT if low bleeding risk (3) DT beyond 12 months after ACS in selected cases (LM lesions, proximal LAD lesions, recurrent MIs, etc.) (4) When using DOAC as part of TT, we may consider lower tested dose of DOAC for stroke prevention in AF		Consider lower INR goal (2–2.5) for patients receiving ASA and P2Y12 inhibitor	Consider lower INR goal (2–2.5) for patients receiving ASA and P2Y12 inhibitor

(III)		Ticagrelor and Prasugrel should not be part of TT			

AF = atrial fibrillation, PCI = percutaneous coronary intervention, ACC/AHA = American College of Cardiology/American Heart association, NSTEMI = non-ST-elevation myocardial infarction, STEMI = ST-elevation myocardial infarction, TT = triple therapy, INR = international normalized ratio, PPI = proton pump inhibitor, GI = gastrointestinal, ACS = acute coronary syndrome, OAC = oral anticoagulant, CAD = coronary artery disease, DT = dual therapy, LM = left main, LAD = left anterior descending, MI = myocardial infarction, DOAC = direct oral anticoagulant, and ASA = aspirin.

**Table 4:** Meta-analyses comparing outcomes of dual with triple therapy.

Study/author	Year	Patient population	Number of patients	Comparison	Results
Agarwal et al. [8]	2017	Patients with an indication for long-term anticoagulation undergoing PCI	7,276	TT versus DT	(1) Less major bleeding with OAC + SAPT (2) Comparable outcomes between OAC + SAPT and TT for MACE, MI, stent thrombosis, CV mortality

Liu et al. [46]	2016	Patients with indication for OAC and undergoing PCI or medically managed ACS	22,842	Network meta-analysis of TT, OAC + C, OAC + A, DAPT	(1) OAC + C had the lowest rate of MACE, CVA, MI, all-cause mortality, and major bleeding

Barbieri et al. [47]	2016	Patients undergoing PCI that required long-term OAC	21,716	TT versus DT	(1) As compared to DT, the use of TT was associated with significant reduction in overall mortality, recurrent MI, and ischemic stroke (2) Patients with TT were found to have a higher incidence of bleeding

D'Ascenzo et al. [48]	2015	Patients with indication for OAC and undergoing PCI or medically managed ACS	7,182	TT versus DAPT, TT versus OAC + C	(1) Major bleeding: DAPT and OAC + C both had less incidence as compared to TT (2) MACE: no benefit of TT over OAC + C or DAPT

Gao et al. [49]	2015	Patients taking OAC with coronary stent implantation	9,185	TT versus OAC + C	(1) Lower incidence of MACE with OAC + C (2) Comparable outcomes between OAC + C and TT for all-cause mortality, MI, ST, ischemic thrombosis, and major and minor bleeding

ACS = acute coronary syndrome; C = clopidogrel; CV = cardiovascular; CVA = cerebral vascular accident; DAPT = dual antiplatelet therapy; DT = dual therapy; MACE = major adverse cardiovascular event; MI = myocardial infarction; OAC = oral anticoagulation; PCI = percutaneous coronary intervention; SAPT = single antiplatelet therapy; TT = triple therapy.
